# Vaccinia virus proteins A36 and F12/E2 show strong preferences for different kinesin light chain isoforms

**DOI:** 10.1111/tra.12494

**Published:** 2017-06-27

**Authors:** William N.D. Gao, David C.J. Carpentier, Helen A. Ewles, Stacey‐Ann Lee, Geoffrey L. Smith

**Affiliations:** ^1^ Department of Pathology University of Cambridge Cambridge UK

**Keywords:** co‐operative binding, cytoskeleton, kinesin light chain, kinesin‐1, microtubule, modulation, vaccinia virus egress

## Abstract

Vaccinia virus (VACV) utilizes microtubule‐mediated trafficking at several stages of its life cycle, of which virus egress is the most intensely studied. During egress VACV proteins A36, F12 and E2 are involved in kinesin‐1 interactions; however, the roles of these proteins remain poorly understood. A36 forms a direct link between virions and kinesin‐1, yet in its absence VACV egress still occurs on microtubules. During a co‐immunoprecipitation screen to seek an alternative link between virions and kinesin, A36 was found to bind isoform KLC1 rather than KLC2. The F12/E2 complex associates preferentially with the C‐terminal tail of KLC2, to a region that overlaps the binding site of cellular 14‐3‐3 proteins. F12/E2 displaces 14‐3‐3 from KLC and, unlike 14‐3‐3, does not require phosphorylation of KLC for its binding. The region determining the KLC1 specificity of A36 was mapped to the KLC N‐terminal heptad repeat region that is responsible for its association with kinesin heavy chain. Despite these differing binding properties F12/E2 can co‐operatively enhance A36 association with KLC, particularly when using a KLC1‐KLC2 chimaera that resembles several KLC1 spliceforms and can bind A36 and F12/E2 efficiently. This is the first example of a pathogen encoding multiple proteins that co‐operatively associate with kinesin‐1.

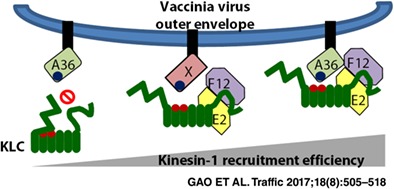

## 1 INTRODUCTION

Microtubule (MT)‐mediated intracellular trafficking is exploited at several stages of the vaccinia virus (VACV) replication cycle.^1^ After de‐envelopment and entry into a host cell, viral cores are transported along MTs^2^ to a perinuclear region where they establish sites of virus replication known as viral factories.^3^ Within these viral factories virus genomes are replicated and packaged into new virions that are surrounded by a single lipid envelope^4^ originating from the endoplasmic reticulum (ER).^5^, ^6^ The majority of these infectious virions, known as intracellular mature virus (IMV), or mature virus (MV) by some authors, remain within the host cell until cell lysis. However, some IMVs are transported on MTs out of virus factories^7^ and become wrapped by 2 additional membranes derived from the early endosomal/trans‐Golgi compartment. These intracellular enveloped virions (IEVs) move along MTs towards the cell surface^8^, ^9^, ^10^, ^11^ from where they are released upon fusion of their outer envelope with the cell membrane. These virions either detach from the cell into the extracellular medium (extracellular enveloped virions, EEV) or remain attached to the cell surface (cell‐associated enveloped virions, CEV) where they trigger a transmembrane signalling cascade that induces actin polymerization, producing actin tails that propel virions away from the host cell (reviewed in Ref 12). Any mutation that reduces IEV formation, IEV egress or actin tail formation has a severe impact on virus spread, resulting in a reduced plaque size in cell culture and attenuated virulence *in vivo*. 13, 14, 15, 16, 17, 18

**Figure 1:**
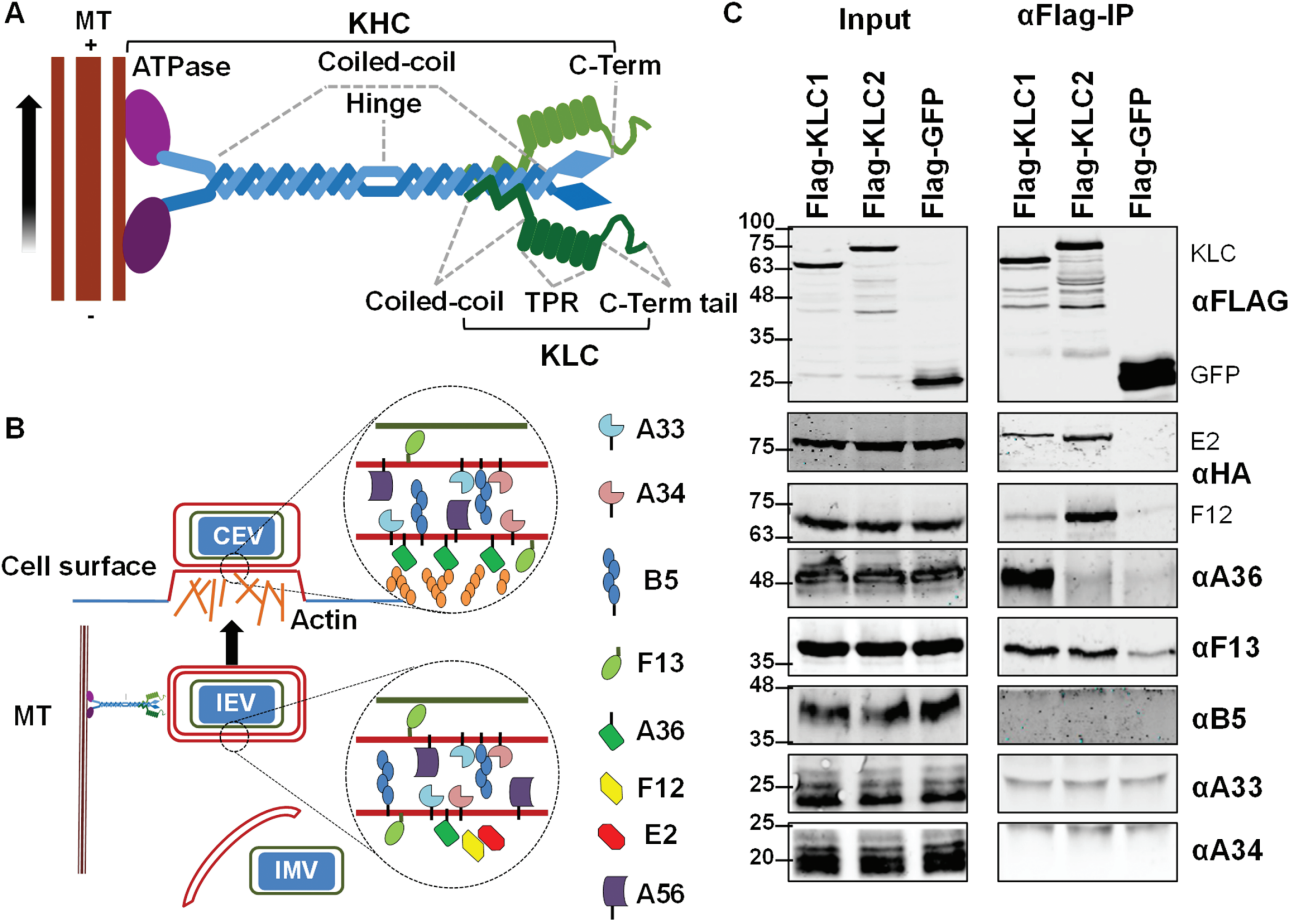
Testing the interaction of VACV IEV proteins with kinesin‐1 by co‐IP with epitope‐tagged KLC1 or KLC2. A, Schematic diagram of the kinesin‐1 complex that mediates trafficking of cargos along microtubules (MT) from the slow‐growing—end to the more dynamic + end oriented towards the cell periphery. Kinesin‐1 is usually represented as a heterotetramer consisting of 2 copies of KHC and 2 copies of KLC. KHC proteins (~110 to 130 kDa) possess an N‐terminal ATPase MT‐binding motor domain (shown in purple), a long coiled‐coil binding domain and C‐terminal tail domain of unknown structure. KLC proteins (51 to 76 kDa depending on the isoform) consist of a short N‐terminal coiled‐coil region responsible for binding to KHC, an α‐helix rich structural region consisting of 6 TPR motifs and a C‐terminal tail. B, The VACV IEV‐associated proteins. During infection some IMV are transported from virus factories on MTs and are wrapped by cellular membranes containing several VACV transmembrane and acylated proteins to form IEV particles. IEVs associate with the kinesin‐1 complex and are transported to the cell surface where F12/E2 dissociate. Virions are externalized by exocytosis and either remain bound to the cell surface as CEVs or are released as EEVs. CEVs can induce the polymerization of actin beneath the CEV particle and this requires the A36 protein. C, Co‐precipitation of IEV proteins with epitope‐tagged KLC1 or KLC2. FLAG‐tagged KLC1, KLC2 and GFP were expressed in HEK293T cells by plasmid transfection. Cells were infected 24 h later with VACV at 5 pfu/cell. Clarified cell lysates were generated 12 h post‐infection (hpi) and used to immunoprecipitate the FLAG‐tagged proteins. The immunoprecipitates were analysed by SDS‐PAGE and immunoblotting. Blots shown are representative of several experiments (*n* = 3) using either vF12‐HA or vE2‐HA.

The movement of IEVs from the site of wrapping to the cell surface is incompletely understood but is mediated by the kinesin‐1 MT‐associated motor complex.^11^ Kinesin‐1, also known as conventional kinesin, is the prototype member of the kinesin protein superfamily.^19^ It consists of a dimer of kinesin heavy chain (KHC) molecules that have 3 isoforms encoded in mammals by the *KIF5A*, *KIF5B* and *KIF5C* genes. Each KHC consists of an N‐terminal MT‐binding ATPase motor domain, an extensive coiled‐coil dimerization domain and a C‐terminal cargo interaction domain (see Figure 1A for a diagrammatic representation). While some kinesin‐1 cargos, such as the mitochondrial associated MIRO‐MILTON complex, interact directly with the KHC C terminus,^20^ many require the presence of 2 copies of the kinesin‐light chain (KLC) adaptor protein. KLCs consist of an N‐terminal coiled‐coil domain responsible for dimerization and KHC interaction, 6 tetratricopeptide repeat (TPR) motifs that each form a helix‐turn‐helix structure, which stack to form a stable protein interaction domain, and a flexible C‐terminal tail (Figure 1A). In mammals 4 isoforms have been identified, each encoded by a separate gene. Both KLC1 and KLC2 are expressed ubiquitously, though KLC1 is often described as being enriched in neuronal cells,^21^ KLC3 is limited to spermatid cells^22^ and KLC4 expression remains to be fully characterized.

At least 6 VACV proteins are directly associated with the wrapping membranes that form the 2 outer envelopes of IEVs (Figure 1B). These include the transmembrane proteins A56,^23^ B5,^18^, ^24^, ^25^ A34,^15^, ^26^, ^27^ A33^15^, ^26^, ^27^ and A36,^16^ and the palmitoylated protein F13.^14^

A36 is associated exclusively with the outer of the 2 IEV envelopes and, upon fusion of this envelope with the plasma membrane, accumulates at the site of CEV attachment.^28^ Here A36 in complex with A33 triggers formation of actin tails.^12^, ^29^, ^30^ The A36/A33 complex is also expressed at the cell surface prior to new virions being made and can induce actin tails beneath superinfecting virions repelling them to enhance virus spread.^31^ A36 is the only protein described to interact directly with kinesin‐1 and link it to IEVs.^32^ A36 possesses a bipartite kinesin‐interaction motif consisting of a tryptophan residue surrounded by 1, 2, or 3 acidic residues (referred to as a WE/D motif), shared by many cellular kinesin‐1 interacting proteins.^33^, ^34^ The structure of a KLC2 TPR domain co‐crystallized with a WE/D motif‐containing peptide (derived from the cellular SifA‐kinesin interacting protein, SKIP) showed binding of the WE/D motif into a groove formed by the second and third TPR motifs on the inner surface of the KLC TPR domain.^35^ A36 probably interacts with KLC in the same manner through its own WE/D motifs.

F12 is a 65‐kDa cytoplasmic protein that, like A36, is associated with IEVs but not IMVs, EEVs or CEVs,^36^ and is required for virus egress.^13^, ^36^ Deletion of F12 results in a more severe egress defect than deletion of A36. However, unlike A36, F12 does not interact directly with the IEV envelope and instead associates with IEVs by binding A36.^37^ E2 is a 86‐kDa cytoplasmic protein and a virus lacking E2 has defects in egress and spread very similar to a virus lacking F12.^34^, ^38^ E2 and F12 form a complex, associate with IEVs during egress and dissociate from virions prior to virus release at the cell surface.^39^ The F12/E2 complex associates with kinesin‐1 through an interaction of E2 with the C‐terminal tail of KLC2.^40^ In the absence of A36 IEVs still undergo MT‐mediated egress.^41^ The ability of F12/E2 to interact with KLC2 may explain how IEVs lacking A36 can move in a MT‐dependent manner, however F12/E2 has not been shown to link kinesin‐1 to IEVs. Alternatively, other link(s) between IEVs and the motor complex mediated by viral or cellular proteins may exist.

In this report, all known VACV‐encoded IEV‐associated proteins (that are absent from IMV particles) were screened by co‐immunoprecipitation for links with kinesin‐1. Unexpectedly, this revealed that during infection A36 interacts almost exclusively with KLC1. This specificity contrasts with the preference of the F12/E2 complex for KLC2.^40^ Biochemical mapping identified the regions of KLCs required for these separate interactions and showed the viral proteins bound to different regions. Lastly, the known interactions of F12 with A36^37^ and F12 with E2^39^ prompted an investigation of whether the F12/E2 complex could enhance the interaction of A36 with KLC. Data are presented showing that the association of A36 with KLC can be enhanced by the presence of F12/E2.

## 2 RESULTS

A yeast‐2‐hybrid screen of the cytoplasmic portions of some of the VACV IEV proteins identified A36 as the only link between IEVs and kinesin‐1.^32^ However, only the TPR region of KLC, the region often associated with cargo interaction,^42^ was tested in this study. A later report that the F12/E2 complex interacted with the C‐terminal tail of KLC^40^ showed that other parts of the KLC protein are also important for cargo interaction and other VACV proteins are involved. Therefore, to test if other IEV proteins interact with KLC, all IEV proteins involved in the formation and egress of IEVs, expressed at endogenous levels during infection, were re‐screened by co‐immunoprecipitation with full‐length KLC.

FLAG‐tagged KLC1 and KLC2 were expressed in HEK‐293 T cells that were infected subsequently with VACV. The FLAG‐tagged KLC was immunoprecipitated from clarified cell lysate and immunoblotted to determine if any of the IEV proteins co‐precipitated (Figure 1C). Antibodies were available for A33, A34, B5, F13 and A36, and so to detect F12 or E2, cells were infected with vF12‐HA (a recombinant VACV expressing HA‐tagged F12^36^) or vE2‐HA (expressing HA‐tagged E2^40^). The results show that A33, A34 and B5 were not co‐precipitated with either KLC. This is consistent with the fact that the majority of these proteins are within the luminal space of the IEV envelope rather than being cytosolic (Figure 1B). In contrast, F12 and E2 were both co‐precipitated with KLC2, confirming previous observations that the F12/E2 complex associates preferentially with the KLC2 isoform.^40^ Optimization of experimental conditions showed that while both F12 and E2 co‐precipitate more efficiently with KLC2, they also co‐precipitate with KLC1 to a lesser degree.

A36 was shown to interact with KLC1 by yeast‐2‐hybrid,^32^ with KLC2 by FRET (Förster resonance energy transfer) microscopy^43^ and both KLC1 and KLC2 by co‐precipitation when overexpressed ectopically.^33^, ^40^ Therefore, it was surprising to find that A36 showed a very strong association with KLC1 and practically no binding to KLC2 (Figure 1C). In many of the previous experiments, A36 was not only overexpressed ectopically but was often expressed in a soluble form lacking its transmembrane domain. The results in Figure 1C represent the first time this interaction has been shown using full‐length A36 expressed at endogenous levels during virus infection.

**Figure 2:**
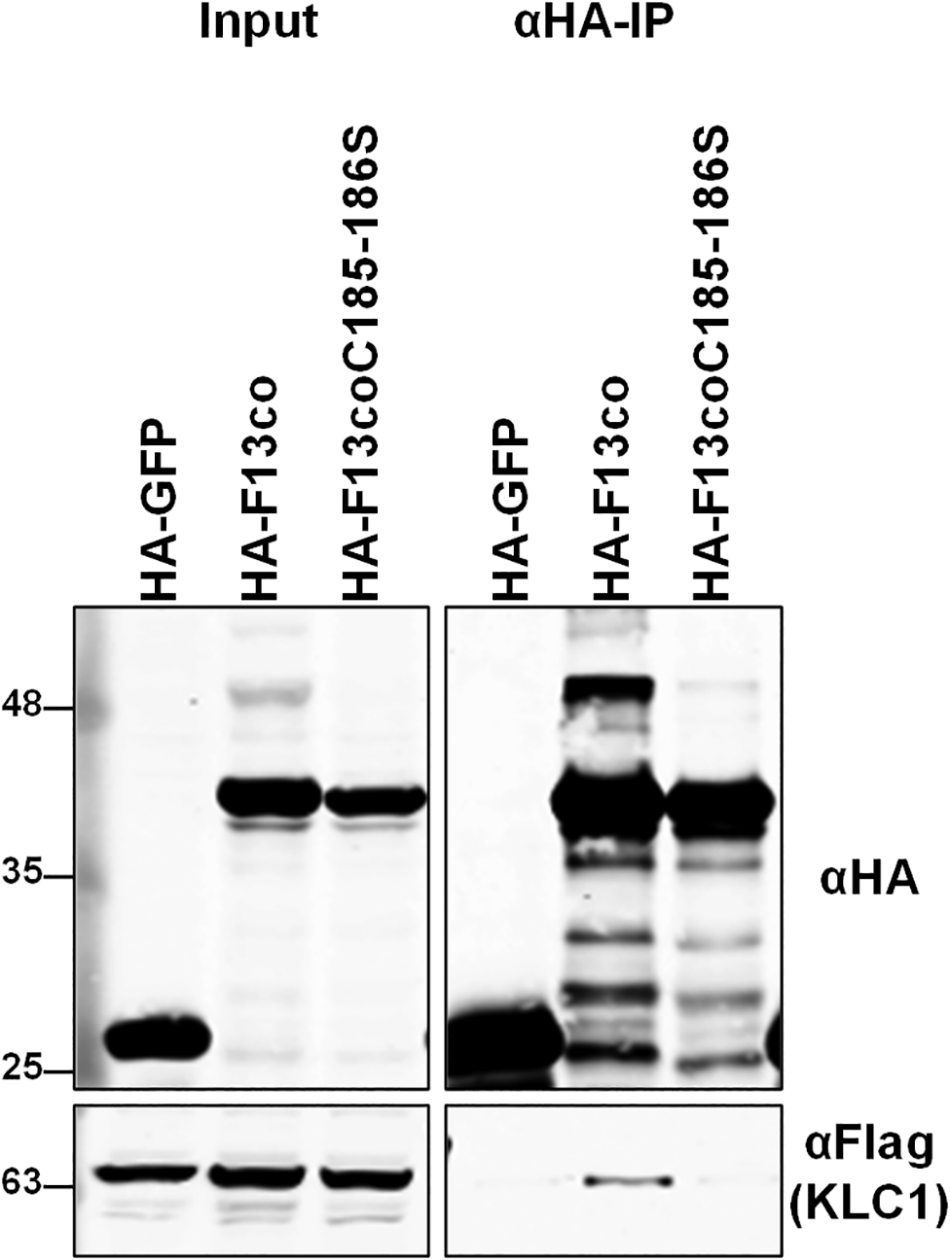
F13 lacking palmitoylation sites fails to associate with KLC. HEK293T cells were co‐transfected with plasmids expressing FLAG‐KLC1 and HA‐F13co, or HA‐F13coC185186S, or HA‐GFP. Cell lysates were harvested 36 h post‐transfection and HA‐tagged proteins were immunoprecipitated and analysed by SDS‐PAGE and immunoblotting with the indicated antibodies. Cell lysates prior to immunoprecipitation (Input) were run in parallel. The immunoblots shown are representative of multiple experiments (*n* = 3).

In addition to A36 and the F12/E2 complex, F13 also co‐precipitated with KLC. Unlike A36, F12 and E2, F13 did not show a KLC isoform specificity, and the levels of F13 co‐precipitating with KLC varied considerably between experiments. To confirm the interaction, the reciprocal co‐immunoprecipitation was attempted with the anti‐F13 antibody, but without success. As an alternative approach, the reciprocal co‐precipitation was then attempted by co‐transfecting HEK‐293 T cells with plasmids expressing HA‐tagged F13 (using a codon optimized allele) along with FLAG‐tagged KLC. Immunoprecipitation of ectopically‐expressed HA‐F13 co‐precipitated FLAG‐KLC (Figure 2). However, the efficiency of co‐precipitation was highly variable and appeared to be sensitive to small differences in detergent concentration. F13 is an abundant IEV protein present between the IMV and IEV inner membrane and on the cytosolic face of the IEV outer membrane (Figure 1B) but is also present in other cellular membranes. F13 does not possess a transmembrane domain and instead associates with membranes via palmitoylation of cysteines 185 and 186.^44^ Mutation of these residues to serine alters F13 localization and abrogates wrapping of IMV to form IEV. Introducing these same mutations into the codon‐optimized HA‐tagged F13 and repeating the co‐precipitation resulted in a loss of KLC co‐precipitation (Figure 2). While this does not discount completely that F13 is a kinesin‐1‐interacting protein (palmitoylation may be required for correct protein folding), taken together with the sensitivity of the co‐precipitation to detergent, these results suggest that the observed F13/KLC interaction is indirect or an artefact of the experimental conditions.

### 2.1 F12/E2 displaces the cellular KLC interacting 14‐3‐3 protein from KLC2

**Figure 3:**
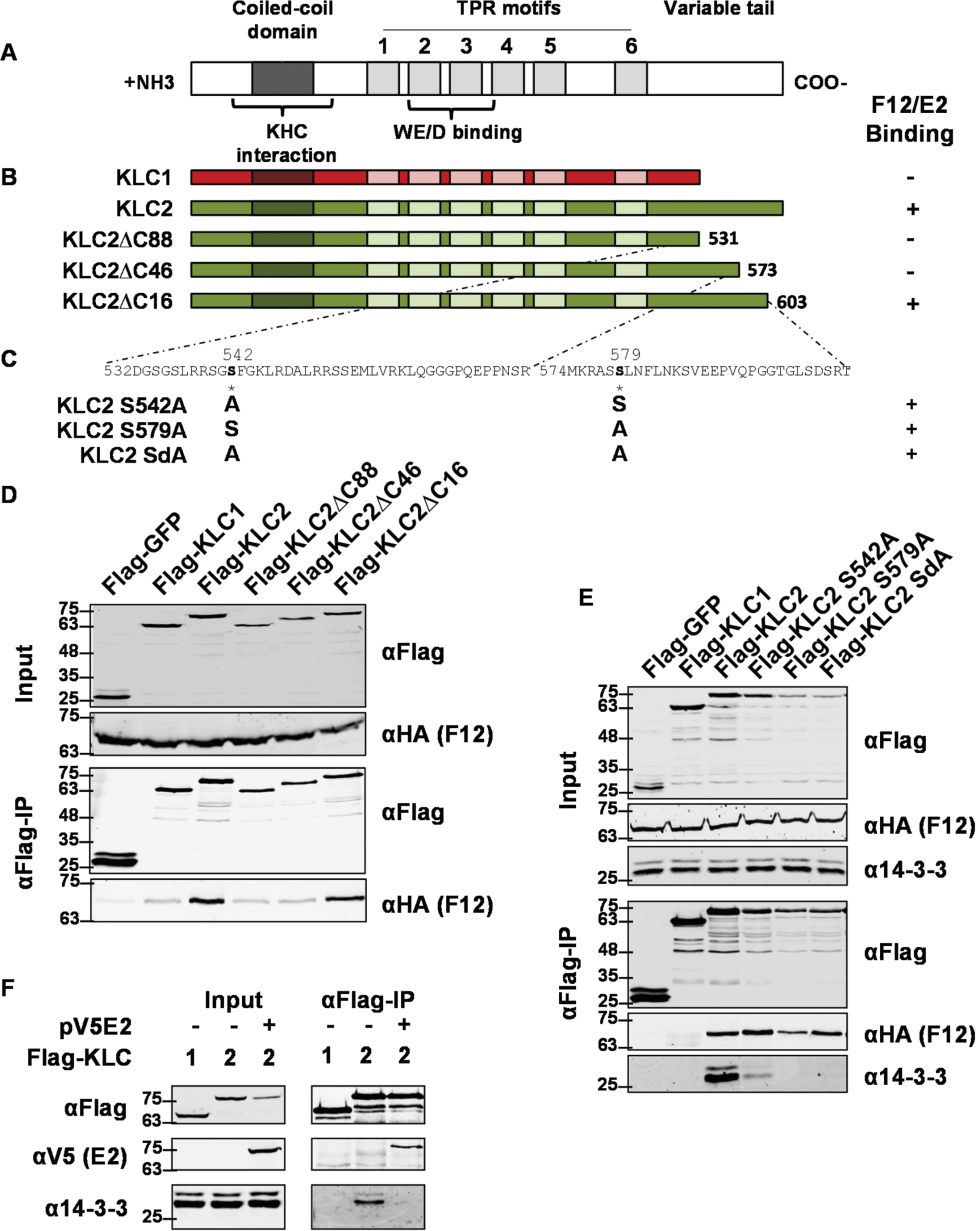
Detailed mapping of the F12/E2 interaction with the KLC2 C‐terminal tail. A, Schematic of KLC structural organization. The coiled‐coil domain, TPR motifs, WE/D interacting domain and C‐terminal variable tail are shown. B, Diagrammatic representation of KLC1 (red), KLC2 (green) and the C‐terminal mutants lacking the last 16, 46 or 88 amino acids. C, Sequence of the KLC2 region required for F12/E2 association. Serines 542 and 579 were mutated to alanine either individually (KLC2 S542A and KLC2 S579A) or together (KLC2 SdA). D, Co‐precipitation of F12 with KLCs. HEK293T cells were transfected with plasmids expressing FLAG‐tagged GFP, KLC1, KLC2 or KLC2 mutants described in panel (B). Cells were infected 24 h after transfection with vF12‐HA at 5 pfu/cell and 12 hpi FLAG‐tagged proteins were immunoprecipitated and analysed by SDS‐PAGE and immunoblotting with the indicated antibodies. Clarified lysates (Input) were analysed in parallel. The interaction of KLCs with F12/E2 is summarized to the right of panel (B). E, Immunoprecipitations and immunoblots were performed as for panel (D) using the mutant KLC2s described in panel (C) and the antibodies shown. The interaction of KLCs with F12/E2 is summarized to the right of panel (C). F, E2 blocks the association of 14‐3‐3 with KLC2. HEK293T cells were co‐transfected with plasmids expressing FLAG‐tagged KLC1 or KLC2 (as indicated) and plasmids expressing E2 or empty vector (EV). Immunoprecipitation and immunoblots were performed as for panel (D) using the antibodies shown. Panels D to F are representative of a minimum of 3 experiments each.

The region of KLC2 responsible for the enhanced association of F12/E2 has been mapped using KLC1/KLC2 chimaeras to the C‐terminal tail of KLC2.^40^ To map this interaction more accurately, a series of mutants of KLC2 were made lacking the C‐terminal 16 (KLC2ΔC16), 46 (KLC2ΔC46) or 88 (KLC2ΔC88) amino acids (Figure 3B). Precipitation of these mutants from cells infected with vF12‐HA showed that only the last 16 amino acids were dispensable for F12/E2 binding and further truncation resulted in F12 co‐precipitation levels equivalent to those with KLC1 (Figure 3D).

To our knowledge, the only other proteins that interact with the C‐terminal tail of KLC2 are the family of cellular 14‐3‐3 proteins^45^ that function as scaffolds involved in the assembly and subcellular localization of signalling complexes.^46^ Binding of 14‐3‐3 to KLC2 is dependent on the phosphorylation of serines 542 and 579^47^ and these serines are present in the region essential for F12/E2 binding. To test if these residues were needed for F12/E2 interaction, FLAG‐KLC2 with serines 542 and 579 mutated to alanines, either individually or together (as shown in Figure 3C) were immunoprecipitated from vF12‐HA‐infected cells. As expected, the mutated KLC2 showed a reduction or loss of 14‐3‐3 co‐precipitation (Figure 3E). However, F12 co‐precipitation was not affected, indicating that F12/E2 association with KLC2 is independent of 14‐3‐3 interaction and does not require serines 542 or 579 to be phosphorylated. Additional evidence that F12/E2 and 14‐3‐3 share overlapping binding sites but do not bind co‐operatively to KLC was provided by the ability of E2 to interfere with the KLC2/14‐3‐3 interaction. In uninfected cells 14‐3‐3 is co‐precipitated with ectopically‐expressed FLAG‐KLC2 (Figure 3 F). When E2 was expressed ectopically in these cells E2 co‐precipitated with FLAG‐KLC2. Expression levels of 14‐3‐3 are not altered in the presence of E2, but it no longer co‐precipitates with FLAG‐KLC2.

### 2.2 Mapping the interaction of A36 to KLC1

**Figure 4:**
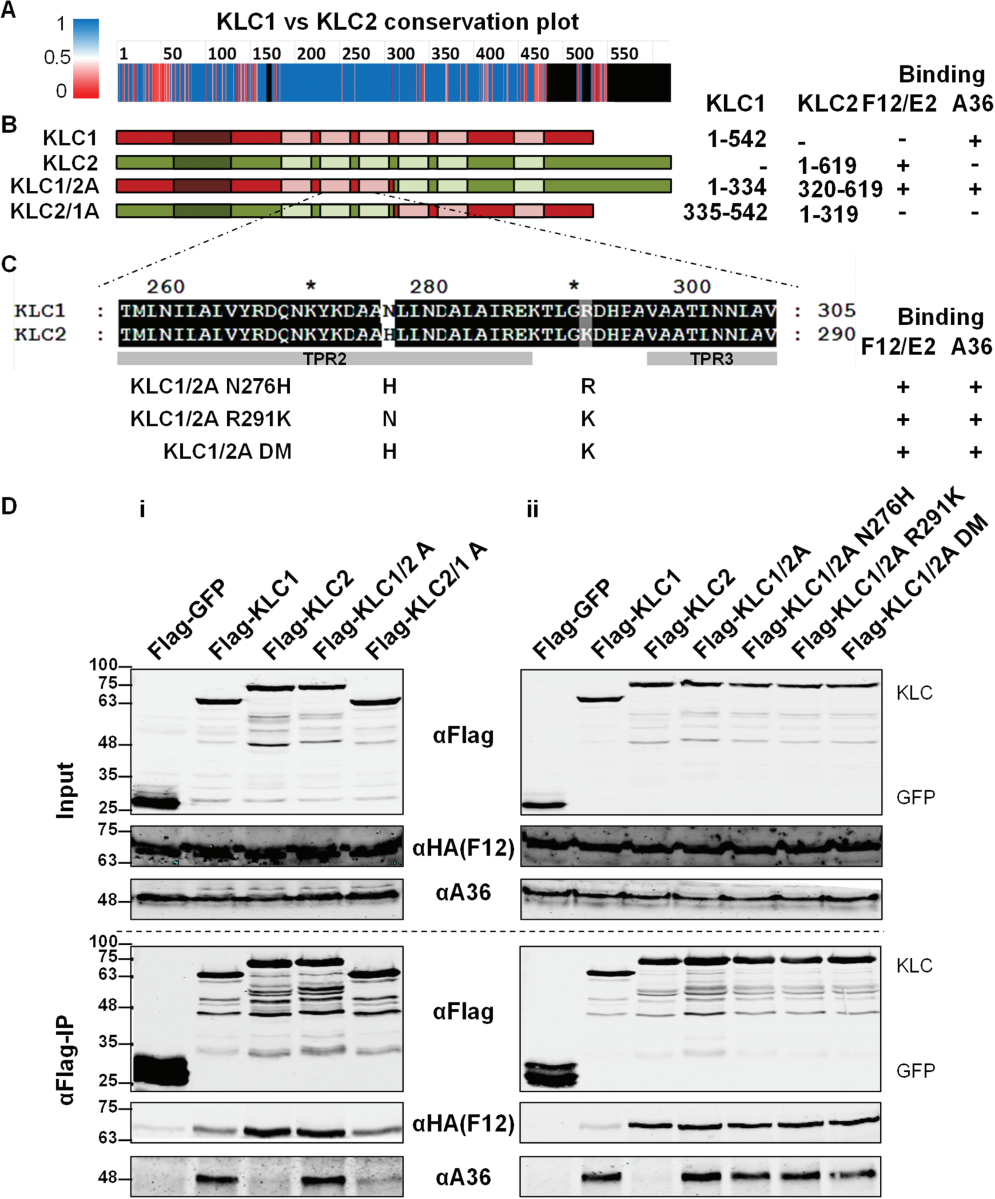
The interaction of A36 with KLC1 is determined by the N‐terminal half of KLC1. A, Conservation plot comparing amino acid conservation scores between KLC1 and KLC2 reproduced from Ref 40 showing conserved amino acid residues in blue, residues that differ between the 2 isoforms in pink and residues that do not align with any region in the other isoform in black. B, Diagrammatic representation of the KLC1 (red), KLC2 (green) and KLC chimaeras. Co‐ordinates of the regions included in each chimaera are given on the right. C, Sequence differences between KLC1 and KLC2 TPRs 2 to 3 and chimaeric KLCs containing these differences. Mutations were introduced into the N‐terminal half of KLC1, changing these amino acid residues to the residues found in KLC2 either individually (KLC1/2A N276H and KLC1/2A R291K) or together (KLC1/2 DM). D, Co‐precipitation of A36 and F12 with the chimaeric and mutant KLCs described in panels (B) and (C) above shown in (i) and (ii), respectively. HEK293T cells were transfected with plasmids expressing FLAG‐GFP, KLC1, KLC2, KLC chimaeras or KLC point mutants. Cells were infected 24 h later with vF12‐HA at 5 pfu/cell. FLAG‐tagged proteins were immunoprecipitated from clarified cell lysates harvested 12 hpi and analysed by SDS‐PAGE and immunoblotting with the indicated antibodies. Clarified lysates (Input) were analysed in parallel. The interaction of KLCs with A36 and F12/E2 is summarized to the right of panels (B) and (C). The blot shown is representative of a minimum of 3 experiments.

KLC1 and KLC2 share a high degree of amino acid similarity, particularly within TPRs 1, 2 and 3 (Figure 4A). To establish what part of the KLC molecule determined A36 binding specificity, co‐precipitations were undertaken using the chimaeric KLC1/KLC2s used to map the F12/E2 interaction.^40^ (Figure 4B). F12 co‐precipitated efficiently with chimaeras possessing the C‐terminal half of KLC2, while A36 co‐precipitated with chimaeras possessing the N‐terminal half of KLC1 (Figure 4Di). Therefore, the specificity‐determining factor for A36 binding is located in the N‐terminal half of the KLC molecule, which includes the WE/D motif binding groove found in TPRs 2 and 3. Within TPRs 1 to 3 KLC1 and KLC2 differ only at amino acids 276 and 291 (Figure 4A and Figure 4C). Neither of these amino acids are located near the WE/D motif binding groove (291 is part of the loop connecting TPRs 2 and 3, and 276 is located on the molecular surface opposite the one that forms the binding groove). Chimaeras in which one or both of these amino acids were mutated from the residue present in KLC1 to the residue present in KLC2 (Figure 4C) were tested for interaction with A36 but neither mutation altered KLC interaction with A36 (Figure 4Dii).

**Figure 5:**
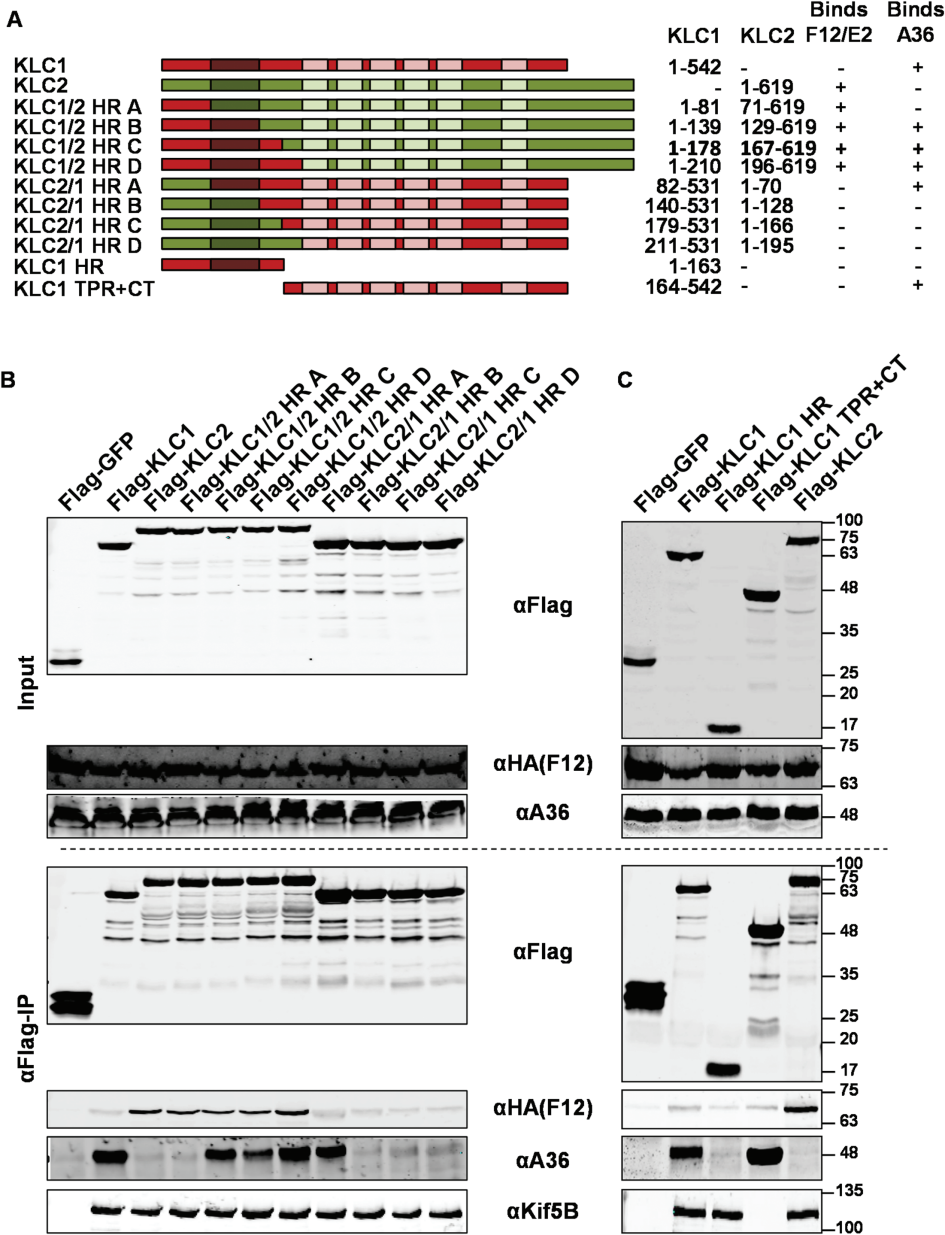
Detailed mapping of the A36/KLC1 interaction. A, Diagrammatic representation of KLC1 (red), KLC2 (green) and KLC chimaeras used. Plasmids were constructed expressing chimaeric KLCs in which varying lengths of the N‐terminal portion containing the heptad repeats (HR) were exchanged between KLC1 and KLC2. KLC1 truncations were constructed expressing either the N‐terminal HR or the TPR and C‐terminal tail of the protein. Co‐ordinates of the amino acid residues of each protein included in the different chimaeras are given on the right. B, Co‐precipitation of A36 and F12 with the chimaeric KLC described in panel (A). HEK293T cells were transfected with plasmids expressing FLAG‐tagged GFP, KLC1, KLC2 or KLC chimaeras. Cells were infected 24 h after transfection with vF12‐HA at 5 pfu/cell. FLAG‐tagged proteins were immunoprecipitated from clarified cell lysates harvested 12 hpi and analysed by SDS‐PAGE and immunoblotting with the indicated antibodies. Clarified lysates (Input) were analysed in parallel. The interaction of KLCs with A36 and F12/E2 is summarized to the right of panel (A). C, Co‐precipitation of A36 and F12 with the truncated KLC1s described in panel (A). HEK293T cells were transfected with plasmids expressing FLAG‐tagged GFP, KLC1, KLC2 or KLC1 truncations. Cells were infected 24 h after transfection with vF12‐HA at 5 pfu/cell. FLAG‐tagged proteins were immunoprecipitated from clarified cell lysates harvested 12 hpi and analysed by SDS‐PAGE and immunoblotting with the indicated antibodies. Clarified lysates (Input) were analysed in parallel. The interaction of KLCs with A36 and F12/E2 is summarized to the right of panel (A). The images shown in panels (B) and (C) are representative of a minimum of 3 experiments each.

This indicated that the specificity for the A36 KLC interaction was upstream of the TPRs. To pinpoint the exact region responsible, additional chimaeric KLCs were generated in which parts of the N‐terminal region were switched between KLC1 and KLC2 (Figure 5A). Only chimaeras possessing the heptad repeat region from KLC1 were able to bind A36 and switching any other region of KLC did not affect A36/KLC co‐precipitation (Figure 5B). As expected, all of the chimaeric KLCs retained their ability to bind to KHC. Subsequently, KLC1 truncations were generated containing either the N‐terminal HR region (KLC1 HR) or TPR domain with the C‐terminal tail region (KLC1 TPR + CT) and tested for their ability to bind A36. Only KLC1 TPR + CT was able to interact with A36, while the HR region on its own was unable to bind A36 (Figure 5C).

### 2.3 The F12/E2 complex is an enhancer of A36/KLC binding

**Figure 6:**
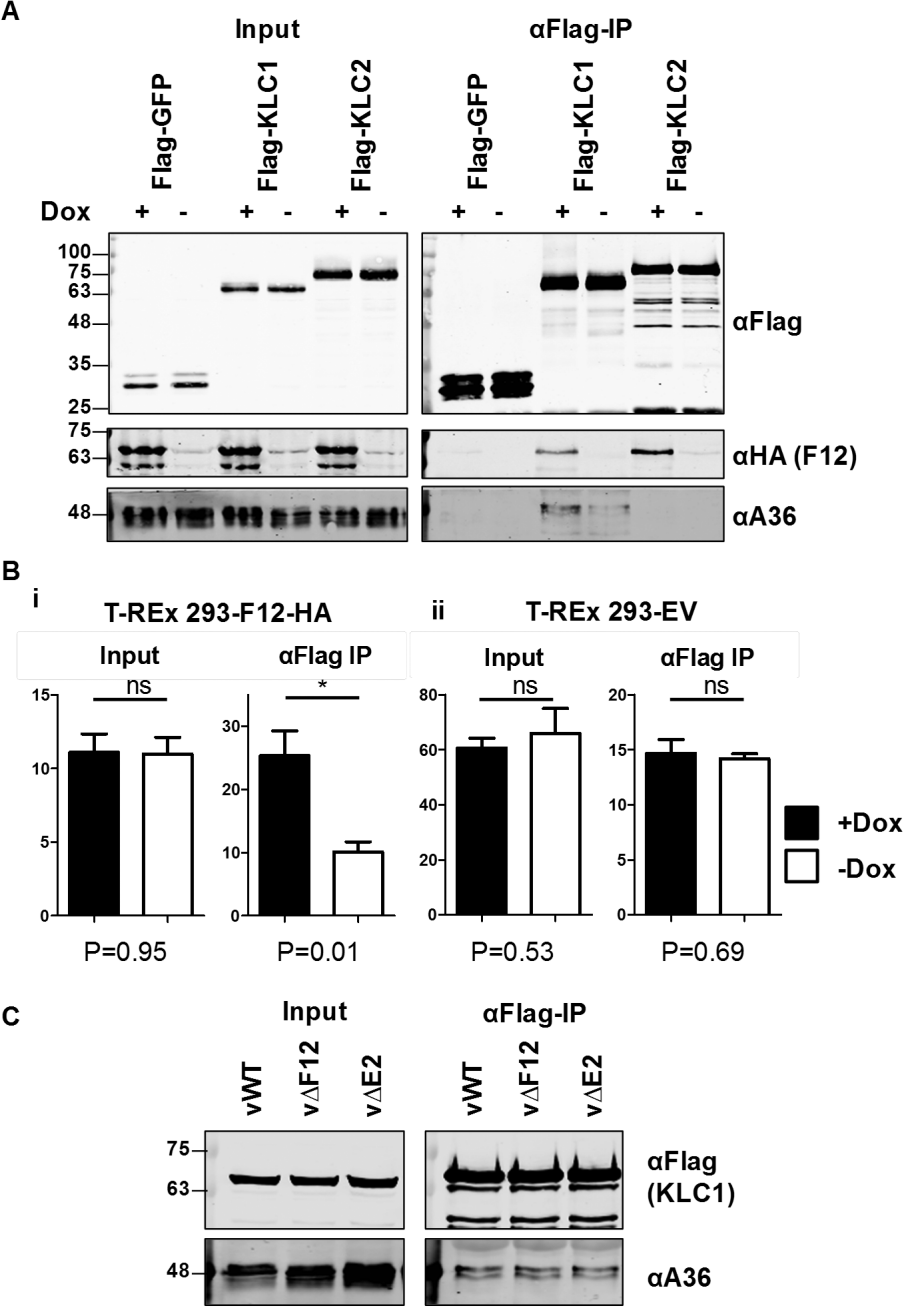
Overexpression of F12 enhances association of A36 with KLC1. A, Co‐precipitation of A36 and KLC1 in the presence or absence of F12. The T‐REx‐293‐F12‐HA cell line was transfected with FLAG‐GFP, KLC1 or KLC2 and infected 32 h post‐transfection (hpt) with vΔF12 at 5 pfu/cell. At the time of infection cells were either induced by addition of doxycycline (final concentration 0.5 µg/ml), or left not induced as indicated. FLAG‐tagged proteins were immunoprecipitated from clarified cell lysates harvested 14 hpi and analysed by SDS‐PAGE and immunoblotting with the indicated antibodies. Clarified lysates (Input) were analysed in parallel. B, Quantification of A36 immunoblot band intensity. The experiment described in panel (A) was repeated with multiple replicates of each sample and anti‐A36 immunoblot band intensities were quantified by LI‐COR scanner (primary data and integrated intensity measurements are shown in Figure S1). Each graph represents the average of quadruplicate samples transferred to the same blotting membrane. *Y*‐axis values are in arbitrary units reflecting relative intensities of bands on the same blots. *P*‐Values calculated by student *t* test comparing each + Dox with each ‐ Dox sample are given underneath the graphs. Graphs are representative of 3 experiments carried out in the T‐REx 293‐F12‐HA cell line and 2 experiments in the T‐REx 293‐EV control cell line. C, Co‐precipitation of A36 and KLC1 from cells infected with WT VACV (vWT), or viruses lacking expression of either F12 (vΔF12) or E2 (vΔE2). HEK293T cells were transfected with FLAG‐KLC1 and infected with the above viruses 24 hpt at 5 pfu/cell. FLAG‐tagged proteins were immunoprecipitated from clarified cell lysates harvested 14 hpi and analysed by SDS‐PAGE and immunoblotting with the indicated antibodies. Clarified lysates (Input) were analysed in parallel.

**Figure 7:**
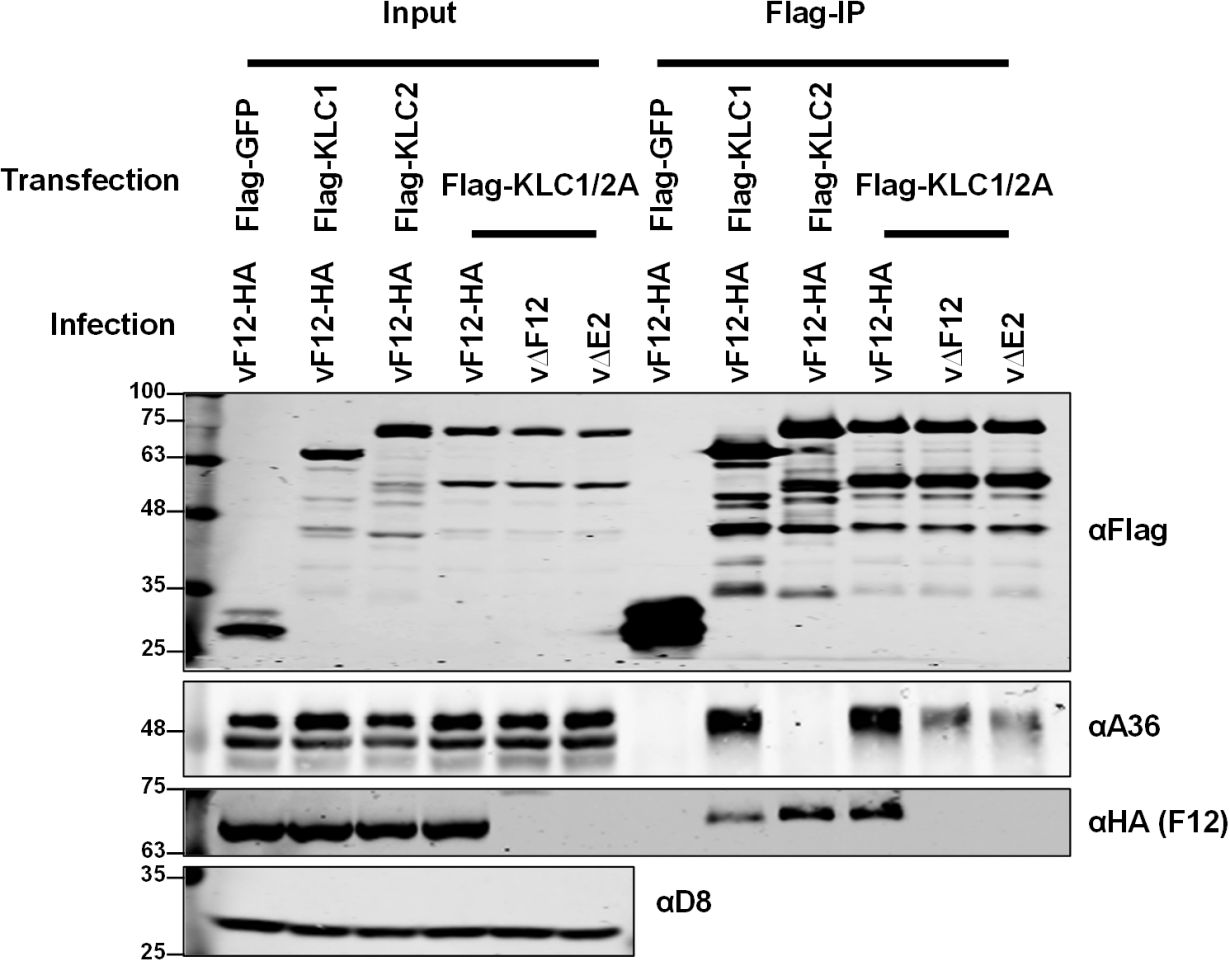
A36 interaction with KLC is reduced in the absence of F12 or E2. Co‐precipitation of A36 and the chimaeric KLC1/2 A in the presence or absence of F12 or E2. HEK293T cells were transfected with FLAG‐GFP, KLC1, KLC2 or KLC1/2 A and infected 24 hpt with vF12‐HA, vΔF12 or vΔE2 at 5 pfu/cell. FLAG‐tagged proteins were immunoprecipitated from clarified cell lysates harvested 14 hpi and analysed by SDS‐PAGE and immunoblotting with the indicated antibodies. Clarified lysates (Input) were analysed in parallel.

Previously, it was hypothesized that the F12/E2 complex may act as an enhancer of the A36/KLC interaction either by stabilizing the interaction or by inducing a conformational shift in KLC promoting A36 interaction. This hypothesis was tested using a cell line expressing F12 inducibly (T‐REx 293‐F12‐HA) and that was transfected with FLAG‐KLC1 and subsequently infected with vΔF12. The levels of A36 that co‐precipitated with KLC1 were higher when F12 was present (Figure 6A). Quantification of repeated experiments confirmed this F12 enhancement of A36 co‐precipitation with KLC1 (Figure 6Bi). Doxycycline treatment of cells did not alter the A36 expression levels (see input samples ± Dox in Figure 6Bi and Figure 6Bii) nor did it increase A36/KLC binding when a control cell line not expressing F12, generated using an empty vector, was used (T‐REx 293‐EV, Figure 6Bii). The experiment was repeated during infection using either wt VACV or mutants lacking F12 (vΔF12) or E2 (vΔE2), but under the conditions tested no change in KLC1/A36 interaction was seen (Figure 6C). However, virus infection of cells expressing a KLC1/2 chimaera (Flag‐KLC1/2 A) that bound both A36 and F12/E2, so that all components may be present in the same complex, showed reduced A36‐KLC interaction in the absence of F12 or E2 (Figure 7B). This suggests that there is co‐operativity in the association of the different components of the IEV trafficking complex, especially if all components are able to efficiently associate.

## 3 DISCUSSION

VACV proteins A36, F12 and E2 all associate with kinesin‐1 during IEV egress and influence IEV egress efficiency. Of these proteins only A36 is associated directly with the IEV particle via a transmembrane domain, yet deletion of A36 is less detrimental to virus egress than deletion of either F12 or E2, and IEVs lacking A36 are still transported in a MT‐dependent manner.^41^ The interaction of A36 with KLC was found initially using a yeast‐2‐hybrid screen that tested the cytoplasmic portions of several IEV proteins (A33, A34, A36, B5 and F12) for binding to the KLC TPR domain.^32^ Here another screen was undertaken using full‐length KLCs and the more physiologically relevant condition of virus‐infected cells where full‐length IEV proteins were expressed at natural levels (Figure 1). This co‐precipitation screen identified A36 and F12/E2 as expected, but also F13. However, the F13 interaction was variable and the observation that a mutant F13 that was unable to associate with membranes was unable to interact with KLC suggested that the F13/kinesin‐1 interaction was either non‐specific or indirect, perhaps mediated by another protein, possibly of cellular origin.

The co‐precipitation screen confirmed the previous report that F12/E2 bound KLC2 preferentially^40^ and mapped the interaction to a region that overlaps the region required for association with the cellular 14‐3‐3 scaffold protein.^47^ The 14‐3‐3 protein was thus a candidate to mediate the interaction of F12/E2 with KLC. However, phosphorylation of KLC2 is needed for 14‐3‐3 interaction,^45^ and serine to alanine mutations that prevented phosphorylation and 14‐3‐3 interaction did not affect F12/E2‐KLC2 interaction.

Surprisingly, the co‐precipitation screen showed A36 had a strong preference for KLC1 rather than KLC2. Past studies looking at A36/KLC binding have reported interactions with both KLC1 and KLC2^32^, ^33^, ^40^, ^43^ but many of these studies used ectopic expression of A36, often using a truncated form of A36 lacking the transmembrane domain. Some studies were also limited to using the TPR domain of KLC. Using full‐length KLCs and a full‐length A36 expressed at natural levels during infection, the interaction was specific for KLC1 and practically absent from KLC2. Additionally, we found that this interaction not only involves the TPR region but also the heptad repeat of KLC, which plays a modulatory role rather than directly mediating the interaction. This is the first example, to our knowledge, that the HR region of KLCs can modulate cargo interactions.

The processes that convert ATP hydrolysis by the kinesin motor into processive motion along MTs has been elucidated in detail.^48^ How the activity of the kinesin complex is regulated and how it interacts with its cargos is less well understood. The regulation of kinesin activity is essential. In its absence, kinesin‐1 motor complexes would be constitutively active resulting in rapid depletion of cellular ATP, and kinesin‐1 redistribution away from where it is needed. Kinesin‐1 regulation relies heavily on autoinhibition.^49^ When not bound to MTs KHC dimers adopt a folded conformation positioning the C‐terminal domain so that it can exert an inhibitory effect on the motor domain ATPase activity.^50^ KLCs stabilize this conformation and prevent the motor domain from binding to MTs.^51^ Binding of cargo proteins to KLC^42^, ^52^ or to the C‐terminal tail of KHC^53^, ^54^ free the motor domain to bind to MTs and mediate movement. In some cases co‐operative binding of several cargo proteins is required to activate kinesin‐1 activity.^42^, ^54^ VACV regulation of kinesin‐1 activity is not yet understood, however it is exploited for egress of IEVs, a process critical for virus spread and virulence, as viruses lacking A36 or F12 are greatly attenuated.^13^, ^16^ The membranes that wrap IMV to form IEV are located near the MT‐organizing centre and contain A36, other VACV‐encoded transmembrane proteins and cellular proteins^55^ that likely could act as cargos for kinesin‐1. If the virus did not modulate kinesin‐1 activity efficiently these membranes might be redistributed to the cell periphery and, consequently, prevent efficient IMV wrapping and IEV egress. Therefore, it is imperative that VACV modulates kinesin‐1 activity, preventing its activation until IEV particles have formed completely.

In this study, the F12/E2 complex is shown to enhance A36 association with KLC despite the fact that A36 and F12/E2 bind preferentially to different KLC isoforms. However, the F12/E2 specificity is not absolute and F12/E2 can also associate with KLC1, and this may explain the enhancement of A36‐KLC1 association when F12 was over‐expressed (Figure 6). Using a chimeric KLC1/2 molecule that bound A36 and F12/E2, a reduction in A36 binding to KLC was observed in the absence of F12 or E2 (Figure 7). Over 16 different KLC1 spliceforms have been described in humans and mice, each with identical heptad repeat and TPR domains but differing in the length and makeup of their C‐terminal tail.^56^ Some of these alternative C‐terminal tails share amino acid sequence similarity with KLC2. The cellular 14‐3‐3 protein, whose KLC interaction overlaps that of F12/E2, can associate with KLC1 spliceform J.^45^ It is therefore conceivable that a KLC1 spliceform exists with which F12/E2 and A36 can bind co‐operatively and may be the actual target of VACV kinesin‐1 engagement.

**Figure 8:**
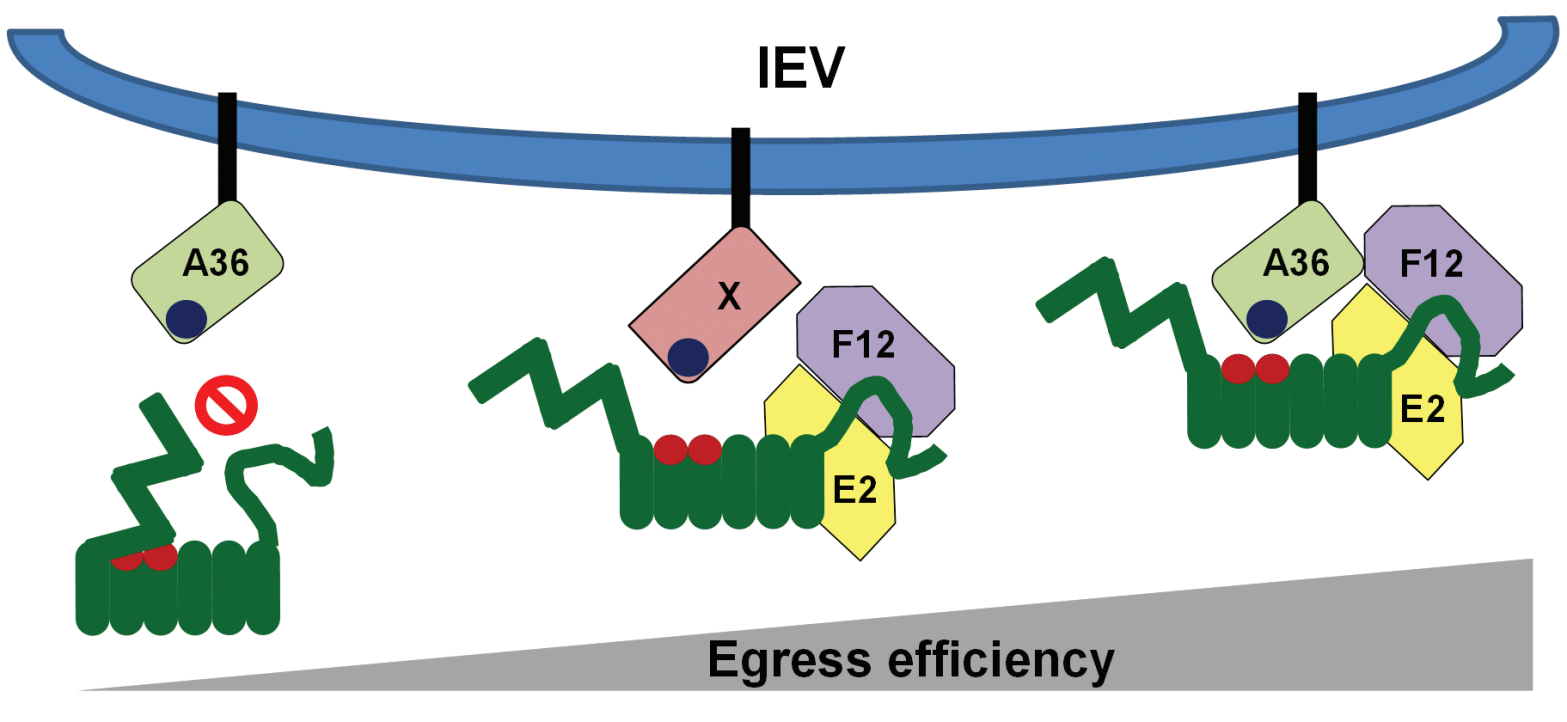
Diagrammatic model of VACV trafficking complex KLC interaction. The outer envelope of IEV particles contain both viral and cellular proteins, some of which possess kinesin interaction motifs such as the WE/D motif (dark blue) present in A36 (light green). In its inactive state KLC exists in a folded conformation with its N‐terminal domain folded over the TPR binding groove for the WE/D motif (Red), preventing association of cargo and activation of kinesin‐1‐mediated trafficking. The VACV F12/E2 complex binds the KLC C‐terminal tail and might induce a conformational change in KLC, increasing accessibility of the TPR binding groove. In the absence of A36, this could lead to recruitment of KLC by any protein with a WE/D motif. Egress is most efficient when both F12/E2 and A36 are present, possibly because the A36/KLC interaction is stabilized by the F12/A36 interaction previously reported,^37^ or because the KLC isoform specificities of A36 and F12/E2 combine to recruit a subset of KLC that targets egress to the correct subcellular domain for efficient virus spread.

The interaction of cargo with KLC is itself regulated by autoinhibition.^57^ A negatively‐charged region surrounding a leucine‐phenylalanine‐proline (LFP) motif, located between the heptad repeat and the first TPR, associates with the TPR‐binding groove in competition with WE/D‐containing peptides. Inactive KLC thus exists in a folded conformation with its C‐terminal tail located such that it could stabilize this folded conformation. Modulatory proteins could either stabilize this conformation or induce a conformational shift facilitating dissociation of the LFP‐containing peptide from the WE/D binding groove. On its own A36 association with KLC is inefficient and may require F12/E2 to provide access to the WE/D binding groove. This model (shown in Figure 8) may explain the differences seen when different components of the IEV trafficking complex are absent. When F12 or E2 are absent the autoinhibited form of KLC cannot be relaxed, blocking binding of A36 and preventing practically all IEV egress. In the absence of A36, F12/E2 can still bind to the KLC C‐terminal tail, relaxing its autoinhibition, allowing other viral or cellular proteins present to associate with the WE/D binding groove. Thus, in the absence of A36 some IEV egress, albeit at reduced efficiency, could occur. This is consistent with the reduced efficiency and run length of IEV on MTs seen in viruses lacking A36.^41^

In this study, the interaction of the VACV IEV trafficking complex with KLC has been mapped. Data presented show that A36 and the F12/E2 complex associate preferentially with different KLC isoforms and with non‐overlapping regions of KLC. It is unknown whether IEVs require KLC1, KLC2 or both for egress, or if they require a particular spliceform or subset of spliceforms. Previous studies in which KLC1 and KLC2 were knocked‐down by siRNA were inconclusive^40^ and knockout cell lines generated using CRISPR based techniques might be useful to test their requirement. The different properties of the various KLC isoforms and spliceforms remains poorly understood. Some cargo‐binding proteins, including cellular TPR‐interacting proteins, display preferential binding of specific isoforms.^33^ Small differences in the KLC C‐terminal tails, such as those present in the different KLC1 spliceforms, can alter the subcellular localization and cargo interaction of KLCs.^58^ The reason for targeting a specific KLC iso/spliceform subset could therefore be to utilize efficiently those motor complexes that are available, or to use motors that will take virions to particular locations on the cell surface.

The role of kinesin‐1 in virion egress has been studied most extensively in members of the *Baculoviridae*, *Herpesviridae* and *Poxviridae*. 59, 60, 61 Each virus family possesses several proteins that associate with kinesin‐1, either by binding KHC (as is the case for herpes simplex virus [HSV] tegument proteins pUS9 and pUS11^62^, ^63^) or by associating with KLC (VACV A36, F12 and E2, and as has been suggested for alphaherpesvirus pUL36 (VP1/2), which possesses putative WE/D motifs^64^ and is required for pseudorabiesvirus MT‐mediated egress^65^ and, together with pUL37, for HSV MT‐mediated egress^66^). The list of cellular proteins that interact with the kinesin‐1 complex is growing steadily. Interestingly, these proteins seem to associate with a range of binding sites located on the KLC TPR region, KLC C‐terminal tail or KHC C‐terminal region. To our knowledge, this report is the first example of the KLC heptad repeat having an influence on cargo interaction with the TPR domain. This report is also the first example of co‐operative binding by one component that associates with the inner groove of the TPR domain and one that binds the C‐terminal tail, and is the first example of co‐operative binding to KLC by any virus. This study furthers our understanding of the mechanisms used by both viruses, and by comparison, cells to modulate cargo interaction with, and trafficking by kinesin‐1.

## 4 MATERIALS AND METHODS

### 4.1 Plasmids

Plasmids expressing FLAG‐tagged murine KLC molecules (pFlag‐KLC1 and pFlag‐KLC2) were a gift from Prof Chris Miller (Kings College London, UK) and have been described.^40^, ^67^, ^68^ Truncated versions of the KLC2 open reading frame (ORF) were generated by PCR amplification (using forward primer 5′‐GATCG AATTC ATGGA CTACA AAGAC GATGA CGAC‐3′ paired with 5′‐GATCT CTAGA CCCAC TCCAC TCAGC TGC‐3′ to generate an amplicon lacking the last 88 amino acids, with 5′‐GATCT CTAGA CCTAG AGTTA GGGGG CTCCT‐3′ for an amplicon lacking the last 46 amino acids, and with 5′‐GATCT CTAGA AGTGC GGCTG TCAGA AAGA‐3′ for an amplicon lacking the last 16 amino acids). These amplicons were cloned into the *Eco*RI‐*Xba*I sites of pFlag‐KLC2 (replacing the full‐length KLC2 ORF). Truncated versions of the KLC1 ORF were generated by PCR amplification (for KLC1 HR forward primer 5′‐GATCG AATTC ATGGA CTACA AAGAC GATGA CGAC‐3′ and reverse primer 5′‐GATCT CTAGA CTAAT CAGAG TCTTT GTCCT CCGA‐3′, for KLC1 TPR + CT forward primer 5′‐GATCG AATTC ATGGA CTACA AAGAC GATGA CGACA AGTCT TCCAA AGAGC CGTTG GAT‐3′ and reverse primer 5′‐GATCT CTAGA CTAGG CTTCC TCCCC TCCG‐3′). These amplicons were cloned into the *Eco*RI‐*Xba*I sites of pFlag‐KLC1 (replacing the full‐length KLC1 ORF).

Mutants of KLC2 in which serines 542 and 579 were mutated to alanines either individually (S542A and S579A) or together (SdA), were generated by site directed mutagenesis using oligonucleotides S542A forward (5′‐GCGCA GTGGC GCCTT TGGGA AGCTC CGGGA TGCTC TGAGA CGCAG CAGTG AGATG C‐3′), S542A reverse (5′‐GCTTC CCAAA GGCGC CACTG CGCCG CAGAG AGCCG CTGCC GTCCC CAC‐3′), S579A forward (5′‐TGAAG AGGGC CAGCG CTCTT AACTT CCTTA ACAAG AGTGT GGAAG AGCCA GTCCA GCCTG GAGGC‐3′) and S579A reverse (5′‐CTTGT TAAGG AAGTT AAGAG CGCTG GCCCT CTTCA TCCTA GAGTT AGGGG GCTCC TGTGG GCCCC‐3′).

Generation of plasmids expressing chimaeric KLC molecules (pFlag‐KLC1/2A and pFlag‐KLC2/1A) have been described.^40^ Site directed mutagenesis using oligonucleotides (N276H forward 5′‐TATAA AGATG CAGCT CACCT CCTGA ACGAC G‐3′, N276H reverse 5′‐CGTCG TTCAG GAGGT GAGCT GCATC TTTAT A‐3′, R291K forward 5′‐AGAAA ACCCT GGGCA AAGAT CACCC CGCGG T‐3′ and R291K reverse 5′‐ACCGC GGGGT GATCT TTGCC CAGGG TTTTC T‐3′) was used to generated KLC1/2A chimaeras in which the amino acids 276 and 291 were mutated from that present in KLC1 to that present in KLC2, either individually (N276H and R291K) or together to generate a double mutant (DM). Additional chimaeric KLCs were generated via splicing by overlap extension,^69^ as described,^40^ using the oligonucleotides listed in Tables S1 and S2, Supporting Information. Chimaera KLC1/2 HR D is identical in sequence to KLC1/2A DM, hence we used KLC1/2A DM as KLC1/2 HR D in Figure 5.

A plasmid expressing N‐terminally V5 (MGKPIPNPLLGLDST)‐tagged codon optimized VACV E2, pcDNA3‐V5E2 (pV5E2) has been described.^40^ A codon‐optimized ORF encoding VACV F13 (GeneArt, Thermo Fisher Scientific, optimized for expression in human cells) was used to replace the E2 ORF in pcDNA3‐HAE2, to generate pcDNA3‐HAF13, a plasmid expressing the F13 protein N‐terminally HA epitope‐tagged. A non‐palmitoylatable version of F13 in which cysteines 185 and 186 were mutated to serines, was generated by site directed mutagenesis using the following oligonucleotides F13coC185‐186S forward (5′‐GCCGC CAGCT CCCTG CCTGT GTCTA CCGCC‐3′) and F13coC185‐186S reverse (5′‐CAGGC AGGGA GCTGG CGGCG CTGCA CAG‐3′).

All site directed mutagenesis was carried out using the QuikChange II site directed mutagenesis kit (Agilent) and all alleles were sequenced to confirm the presence of the mutations.

### 4.2 Cells

RK‐13 cells (rabbit kidney cell line, ATCC CCL‐37) were maintained in minimal essential medium (Gibco) supplemented with 10% FBS and penicillin‐streptomycin (Pen/Strep, Gibco). HEK 293T (human embryonic kidney cell line, ATCC CRL‐11268) and BS‐C‐1 (African green monkey cell line, ATCC CCL‐26) cell lines were maintained in Dulbecco's modified Eagle medium (Gibco) supplemented with 10% FBS and Pen/Strep. A HEK cell line expressing the HA‐tagged VACV protein F12 under the control of a doxycycline‐inducible promoter (T‐REx 293‐F12‐HA) has been described.^40^ The pcDNA4/TO empty vector from the T‐REx inducible expression system (Thermo Fisher Scientific) was used to generate a control cell line (T‐REx 293‐EV) using the same procedure as for the F12‐HA expressing cell line. T‐REx 293 cell lines were maintained in DMEM supplemented with 10% FBS, Pen/Strep, 10 µg/mL blasticidin (Gibco) and 100 µg/mL zeocin (Invitrogen), and induced with 0.5 µg/mL doxycycline when required.

### 4.3 Viruses

All infections were carried out using the Western Reserve (WR) strain of VACV or mutants generated from this strain. Viruses vΔF12, lacking expression of F12,^13^ or vΔE2, lacking expression of E2,^38^ or vF12‐HA, expressing HA‐epitope tagged F12,^13^ or vE2‐HA, expressing HA‐epitope tagged E2^40^ have been described. Viruses were amplified in RK‐13 cells and titrated on monolayers of BS‐C‐1 by plaque assay.

### 4.4 Immunoprecipitations

For immunoprecipitations, cells were seeded in 10‐cm diameter tissue culture dishes. Cells were transfected when they had reached 70% confluence using transit‐LT1 transfection reagent (Mirius). If required cells were infected 24 h later at 5 plaque‐forming units (pfu) per cell. Cell lysates were harvested 12 or 14 h later (36 or 38 h post‐transfection) by scraping into IP wash buffer (50 mM Tris‐HCl pH 7.5, 0.5% [v/v] nonidet‐P40 substitute [Sigma], 150 mM NaCl, 2 mM EDTA) supplemented with cOmplete Mini EDTA‐free protease inhibitor cocktail tablets (Roche). Cells were lysed on ice for 45 min with occasional vortexing and lysates were clarified by centrifugation (15,000*g*, 15 min, 4°C). Epitope‐tagged proteins were then immunoprecipitated from clarified lysates using either anti‐FLAG M2 affinity gel (Sigma) or anti‐HA monoclonal antibody (clone HA‐7)‐conjugated agarose beads (Sigma). Immunoprecipitations were incubated for 4 h or overnight while rotating and then washed 4 times with IP wash buffer. Immunoprecipitated proteins were eluted by boiling in Laemmli SDS‐PAGE loading buffer prior to analysis by SDS‐PAGE and immunoblotting.

Proteins separated by SDS‐PAGE and transferred onto Hybond ECL nitrocellulose membrane (GE Healthcare) were probed with the following commercial antibodies; rabbit polyclonal α‐FLAG (Sigma‐Aldrich, F7425, 1:5000), rabbit polyclonal α‐HA (Sigma‐Aldrich, H6908, 1:1500), rat monoclonal α‐HA (Chromotek, 7C9, 1:1000), mouse monoclonal α‐HA (BioLegend, HA.11, 1:1000), rabbit polyclonal α‐14‐3‐3 (Santa Cruz, sc‐629, 1:1000), rabbit monoclonal α‐KIF5B (Abcam, ab167429, 1:1000), the following antibodies specific for VACV proteins; mouse monoclonal α‐A36 (1:1000),^28^ rat monoclonal α‐F13 (1:1000), rat monoclonal α‐B5 (1:100),^70^ mouse monoclonal α‐A33 (1:5), mouse monoclonal α‐A34,^71^ and mouse monoclonal AB1.1 specific for the VACV protein D8.^16^ Blots were probed with IRDye‐conjugated secondary antibodies (LI‐COR), and imaged using a LI‐COR Odyssey scanner. Detection of the A36 protein, which has a similar molecular mass to the antibody heavy chain, was achieved by using was a Biotin‐SP AffiniPure goat anti‐mouse IgG, light chain specific (Jackson ImmunoResearch, 115‐065‐174). A33 and A34, which have overlapping molecular masses to the antibody light chain, were detected using a biotin‐SP AffiniPure goat anti‐mouse IgG, Fcγ fragment specific (Jackson ImmunoResearch, 115‐065‐071). Biotinylated secondary antibodies were visualized using IRDye‐conjugated Streptavidin (LI‐COR).

For quantitative analysis of relative protein levels, band intensities were measured using the LI‐COR Odyssey scanner software with localized background normalization. Averages were calculated from 3 or 4 replicates per sample and comparisons were made between adjacent samples run on the same gel and transferred onto the same membrane. Statistical analysis was done using GraphPad Prism (version 5) software.

## Supporting information

Editorial Process

**Figure S1.** Quantification of A36/KLC1 interaction ± F12 expression Primary immunoblotting data used for quantifying the level of A36 co‐immunoprecipitation with Flag‐KLC1 in the presence (+) or absence (−) of Doxycycline (Dox) induction either in T‐REx 293‐F12‐HA (A) or T‐REx 293‐EV (B) cells. Values below the blots are integrated intensity (I.I.) measurements (minus local background) of the bands shown and were used to generate the data shown in Figure 6B.
**Table S1.** List of primer sequences.
**Table S2.** Details of primer pairs used to generate KLC1/2 chimaeras by overlap extension.
