# Supplementary material for: Vaccinia virus proteins A36 and F12/E2 show strong preferences for different kinesin light chain isoforms
Source: Traffic. 2017 Jun 27;18(8):505–18. doi: 10.1111/tra.12494 (PMC5519951; doi:10.1111/tra.12494)

Vaccinia Virus Proteins A36 and F12/E2 Show Strong Preferences for Different KLC Isoforms

William N. D. Gao^1¶^, David C. J. Carpentier^1¶^, Helen A. Ewles^1^, Stacey-Ann Lee^1,#^ & Geoffrey L. Smith^1^*

^1^Department of Pathology, University of Cambridge, Tennis Court Road, Cambridge CB2 1QP, UK

^¶^These authors contributed equally

^#^Current address: The Francis Crick Institute, 1 Midland Road, London NW1 1AT, UK

*Corresponding author: Prof. Geoffrey L. Smith, Department of Pathology, University of Cambridge, Tennis Court Road, Cambridge CB2 1QP, UK, email: [gls37@cam.ac.uk](mailto:gls37@cam.ac.uk), tel: +44(0)1223 333692

Supplemental Information

**Supplemental Table 1: List of primer sequences**

| **Primer Name** | **Sequence** |
| --- | --- |
| **WG011** | 5' GAT CGA ATT CAT GGA CTA CAA AGA CGA TGA CGA C |
| **WG014** | 5' GAT CTC TAG ATT AGC CCA CCA GGG AGC TT |
| **WG019** | 5' GAT CTC TAG ACT AGG CTT CCT CCC CTC CG |
| **WG139** | 5' CCC CAA GCC CAA GCT CCA GCA TCT CCA GG |
| **WG140** | 5' TGG AGC TTG GGC TTG GGG AGG CTC AG |
| **WG141** | 5' CCT CCA GCT GCG CCA CCG ACT GCT CG |
| **WG142** | 5' GTG GCG CAG CTG GAG GAA GAG AAG C |
| **WG143** | 5' TGG GAA ACA GAT CAT CCA ACG GCT CTT TGG A |
| **WG144** | 5' CCG TTG GAT GAT CTG TTT CCC AAT GAA GAT GAA CAG |
| **WG145** | 5' CGC TCA GGC CCA GCT CGA TGG CCT CCA G |
| **WG146** | 5' GAG CTG GGC CTG AGC GAG GCG |
| **WG147** | 5' CCT CCA GCT GAG CCA CCG CCT GTT CAC T |
| **WG148** | 5' GGT GGC TCA GCT GGA GGA GGA GAA GAA A |
| **WG149** | 5' TTG GGA AGA GGT CAT CCA GGG AGT CTT TGG G |
| **WG150** | 5' CCC TGG ATG ACC TCT TCC CAA ATG ACG AGG AC |
| **WG151** | 5’ GCA GCC TTG CTG GGA TCT CAT AGC CCC CAT G |
| **WG152** | 5’ AGA TCC CAG CAA GGC TGC GCA CG |

**Supplemental Table 2: Details of primer pairs used to generate KLC1/2 chimaeras by overlap extension**

|  | **5' fragment** | | | **3' fragment** | | |
| --- | --- | --- | --- | --- | --- | --- |
| **Chimera Name** | **Template** | **Primer 1** | **Primer 2** | **Template2** | **Primer 13** | **Primer 24** |
| KLC1/2 HR A | KLC1A | WG011 | WG139 | KLC2 | WG140 | WG014 |
| KLC1/2 HR B | KLC1A | WG011 | WG141 | KLC2 | WG142 | WG014 |
| KLC1/2 HR C | KLC1A | WG011 | WG143 | KLC2 | WG144 | WG014 |
| KLC2/1 HR A | KLC2 | WG011 | WG145 | KLC1A | WG146 | WG019 |
| KLC2/1 HR B | KLC2 | WG011 | WG147 | KLC1A | WG148 | WG019 |
| KLC2/1 HR C | KLC2 | WG011 | WG149 | KLC2 | WG150 | WG014 |
| KLC2/1 HR D | KLC2 | WG011 | WG151 | KLC2 | WG152 | WG014 |

### Supplemental Figure S1: Quantitation of A36/KLC1 interaction ±F12 expression

Primary immunoblotting data used for quantifying the level of A36 co-immunoprecipitation with Flag-KLC1 in the presence (+) or absence (-) of Doxycycline (Dox) induction either in T-REx 293-F12-HA (A) or T-REx 293-EV (B) cells. Values below the blots are integrated intensity (I.I.) measurements (minus local background) of the bands shown and were used to generate the data shown in Figure 6B.


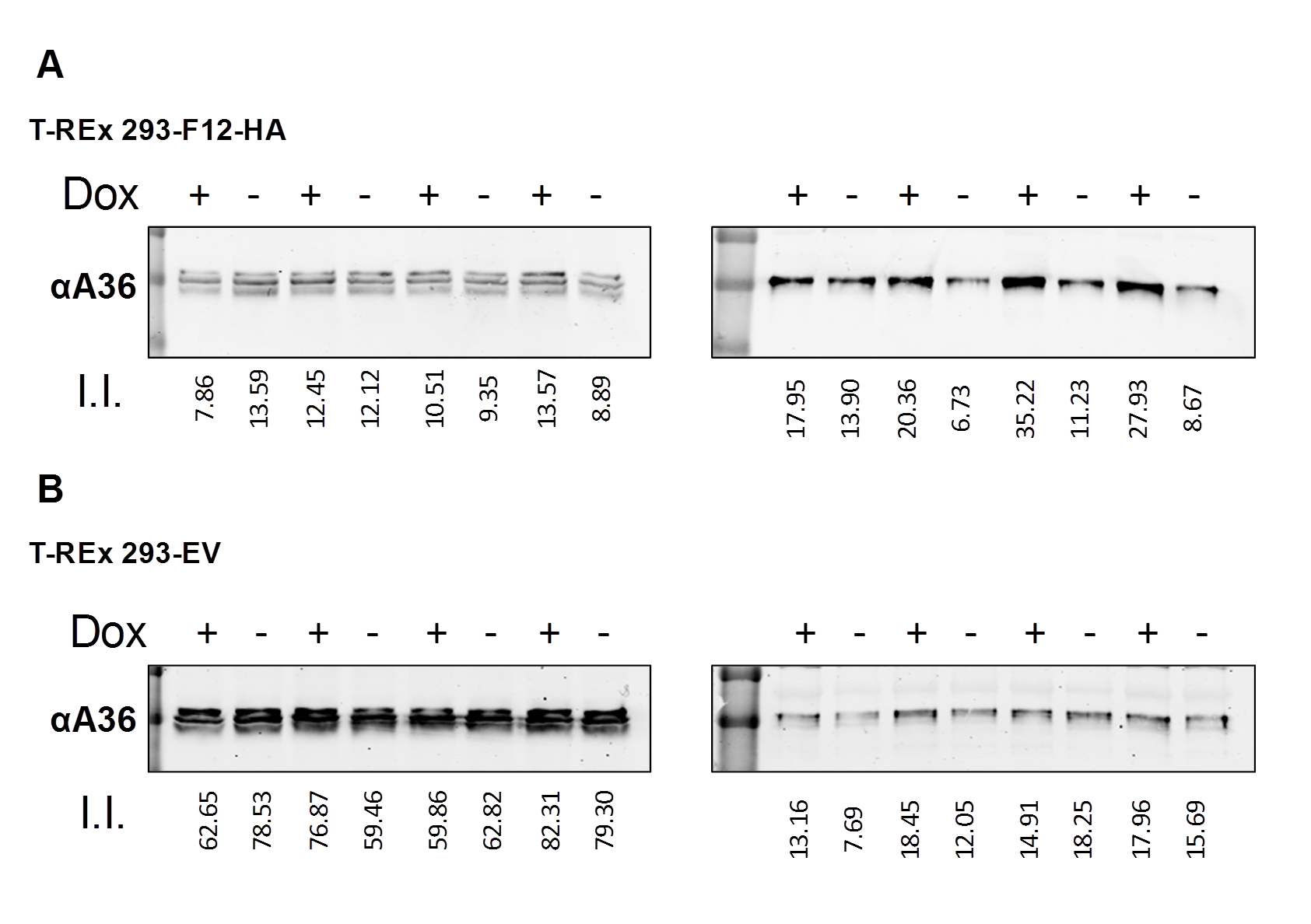

Supplement: Supplementary file 2 — Figure S1. Quantification of A36/KLC1 interaction ± F12 expression Primary immunoblotting data used for quantifying the level of A36 co‐immunoprecipitation with Flag‐KLC1 in the presence (+) or absence (−) of Doxycycline (Dox) induction either in T‐REx 293‐F12‐HA (A) or T‐REx 293‐EV (B) cells. Values below the blots are integrated intensity (I.I.) measurements (minus local background) of the bands shown and were used to generate the data shown in Figure 6B. Table S1. List of primer sequences. Table S2. Details of primer pairs used to generate KLC1/2 chimaeras by overlap extension. [file TRA-18-505-s002.docx]
