# Supplementary material for: Vaccinia virus proteins A36 and F12/E2 show strong preferences for different kinesin light chain isoforms
Source: Traffic. 2017 Jun 27;18(8):505–18. doi: 10.1111/tra.12494 (PMC5519951; doi:10.1111/tra.12494)
Supplement: Supplementary file 1 — Editorial Process [file TRA-18-505-s001.pdf]

Title:	Vaccinia Virus Proteins A36 and F12/E2 Show Strong Preferences for Different KLC Isoforms
Authors:	William N. D. Gao, David C. J. Carpentier, Helen A. Ewles, Stacey-Ann Lee & Geoffrey L. Smith
Article Type:	Original Research

Monitoring Editor	Trina Schroer
Date Submitted	24 February 2017
Date for Decision 1	29 March 2017
Date Resubmitted	18 April 2017
Date for Decision 2	4 May 2017
Accepted	5 May 2017

Decision and Reviews

Dear Dr. Smith,

Thank you for submitting your manuscript entitled "Vaccinia Virus Proteins A36 and F12/E2 Show Strong Preferences for Different KLC Isoforms" to be considered for publication in Traffic. I asked two colleagues who are experts in the field to review the manuscript and their verbatim comments are appended below. Referee 1 is of the view that the work presented in this paper does not provide the kind of mechanistic cell biological advance that we aim for in the papers that are published in Traffic. Referee 2 has a very different opinion, noting that the work you present here provides important insights into the controversy regarding the relative importance of A36 versus F12/E2 complex. Despite the interest of referee 2 in the study in principle, they expressed concern that some of your conclusions are not fully supported by the data presented. Referee 2 also had concerns about the way the data are presented.

I am pleased to tell you that we would be willing to consider a revised version of the manuscript that addresses the concerns of both referees. Given the overall negative impression of referee 1 with regards to the physiological relevance of your findings it is important that you do your best to provide a more persuasive argument. I expect this will be facilitated by specifically by highlighting the novel aspects of the work, as suggested by referee 2. Of course, you will also need to address the full complement of concerns raised by referee 2.

Although Traffic is not able to accept this manuscript for publication at this time, I believe you will be able to address the referees concerns fully and I look forward to receiving a suitably revised manuscript in the near future. To expedite handling when you resubmit please be sure to include a response that details how you have addressed each of the referees' concerns.

Sincerely,

Trina A. Schroer, Ph.D.
Co-Editor

___

Referee's Comments to the Authors

Referee: 1

Comments to the Author

In essence the data show interaction of kinesin light chain 1 and 2 with vaccinia virus proteins. For this the authors over-express FLAG-tagged version of KLC1 and 2 in HEK cells which they subsequently infect with vaccinia virus to show which viral proteins will co-IP with the over-expressed constructs.

The manuscript collects additional observations that should complement this; such as that the 14-3-3 protein is displaced from KLC when viral proteins bind. In a molecular biology approach the region of binding of one viral protein is mapped. Finally, the authors provide some evidence that one viral complex enhances the binding of another protein to KLC.

This reviewer misses 1. A clear story 2. A relevance for the cell or the virus. As such the data are a collection of results that are not within the scope of Traffic and would much better fit a journal with a biochemical focus.
In addition, in some parts the data are not much different from a recent publication of the same group in PLoS pathogens (Carpentier et al., 2015).
All in all therefore I cannot recommend the data for Traffic.

Referee: 2

Comments to the Author
In this manuscript, the authors investigated the interaction of structural proteins of the intracellular enveloped virions of vaccinia virus with kinesin-1, in particular with kinesin light chains KLC1 and KLC2. Previous work from the groups of Michael Way and Geoffrey Smith has already indicated that the vaccinia protein A36 and the vaccinia protein complex F12/E2 operate during microtubule mediated motility of the intracellular enveloped virus form of vaccinia virus. The present manuscript adds to these investigations by providing a thorough characterization of the differences in binding specificity of A36 and F12 for KLC1 or KLC2. The manuscript is overall written, and the data in general support the conclusions drawn.

Specific Comments:
1. This reviewer was lost in the description of Figure 3, particularly Figures 3D and 3E (page 3, right column), and had to read it several times until it became clear what the authors possibly meant. The authors should revise this paragraph for clarification and provide clear links between the main text and the respective figure panels.
2. The data in Figure 6A does not fully support the conclusions. The authors show that upon induction with Dox of the stably transduced cell line T-Rex-292-F12-HA the expression of F12 is induced. Upon infection with a vaccinia virus lacking F12, the authors claim that they co-precipitate a higher amount of A36 with KLC1 upon induction of F12. Based on these data, the authors suggest in the abstract and in Figure 8 that the F12/E2 complex cooperatively enhances the association of A36 with KLC1. However, this conclusion would only be correct if the induction with Dox would not change the expression levels of the Flag-tagged KLC1.
But unfortunately, exactly this part of the blot shown is messed up. If the authors cannot provide a blot with clear data on Flag-tagged KLC1, F12, and A36, the authors need to remove this part of the manuscript. They claim that they have conducted this experiments several times, and even provide a quantitation of such blot data in Fig. 6B. However, without supportive primary data this quantitation is flawed.
Furthermore, without these data, the major conclusion of the manuscript would be lost.

Minor Comments:
1. Reference 64 does not provide data showing that pUL36 of herpes simplex virus interacts with KLC. This comment needs to be removed. However, there is newer data deleting potential KLC binding motifs in pUL36 from the Sodeik group in Germany that should be discussed in this context.
2. From which species are the expression constructs for the FLAG-tagged kinesin-light chains?

Author Rebuttal
Dr Trina A. Schroer,
Editor, Traffic.

Re:
Gao et al.,
Vaccinia virus proteins A36 and F12/E2 show strong preferences for different KLC isoforms

Dear Dr Schroer,

Thank you for sending the comments from the 2 referees and for your covering letter. Our response to the issues raised follows below. For clarity the comments from the referees are shown in "quotes".

Referee: 1

"In essence the data show interaction of kinesin light chain 1 and 2 with vaccinia virus proteins. For this the authors over-express FLAG-tagged version of KLC1 and 2 in HEK cells which they subsequently infect with vaccinia virus to

show which viral proteins will co-IP with the over-expressed constructs.

The manuscript collects additional observations that should complement this; such as that the 14-3-3 protein is displaced from KLC when viral proteins bind. In a molecular biology approach the region of binding of one viral protein is mapped. Finally, the authors provide some evidence that one viral complex enhances the binding of another protein to KLC.

This reviewer misses 1. A clear story 2. A relevance for the cell or the virus. As such the data are a collection of results that are not within the scope of Traffic and would much better fit a journal with a biochemical focus.

In addition, in some parts the data are not much different from a recent publication of the same group in PLoS pathogens (Carpentier et al., 2015).

All in all therefore I cannot recommend the data for Traffic."

We disagree with the negative tone of this referee and note that there was no criticism of any of the experimental data presented. We point out that:

i) This paper provides a comprehensive screen of vaccinia virus proteins that are associated with the intracellular enveloped virus (and absent from intracellular mature virus) for interaction with the kinesin 1 motor. The viral proteins were all expressed as full length proteins during infection (rather than overexpressed fragments outwith infection as described in some studies previously). Such a screen had not been done before and showed that only A36 and the F12/E2 complex were able to bind KLCs.
ii) The paper shows that the F12/E2 complex and A36 protein have very different specificity for KLC isoforms. This specificity of A36 for KLC1 is completely new. We know of no other virus that encodes different proteins that bind to different KLC isoforms. This is without precedent.
iii) The interaction of F12/E2 is fine mapped to the C terminus of KLC2 where it's binding overlaps with and competes with the binding of 14-3-3 protein to KLC2. Therefore, the binding of the complex to KLC2 is independent of 14-3-3.
iv) The paper maps the interaction of A36 to KLC1 to a quite different region of the KLC and also determines the region of KLC1 determining the selective binding.
v) Lastly, the paper demonstrates that the interaction of A36 with KLC is enhanced in the presence of the F12/E2 complex showing co-operativity in this interaction. This is entirely novel.

So there is a very clear message and this is made explicit in the manuscript.

Referee: 2

"In this manuscript, the authors investigated the interaction of structural proteins of the intracellular enveloped virions of vaccinia virus with kinesin-1, in particular with kinesin light chains KLC1 and KLC2. Previous work from the groups of Michael Way and Geoffrey Smith has already indicated that the vaccinia protein A36 and the vaccinia protein complex F12/E2 operate during microtubule mediated motility of the intracellular enveloped virus form of vaccinia virus. The present manuscript adds to these investigations by providing a thorough characterization of the differences in binding specificity of A36 and F12 for KLC1 or KLC2. The manuscript is overall written, and the data in general support the conclusions drawn."

We thank the referee for the positive assessment of our manuscript.

"1. This reviewer was lost in the description of Figure 3, particularly Figures 3D and 3E (page 3, right column), and had to read it several times until it became clear what the authors possibly meant. The authors should revise this paragraph for clarification and provide clear links between the main text and the respective figure panels."

The confusion may result in part from our failure to refer to Figure 3E in the text and this omission has been corrected. We are grateful for the opportunity to do this. In addition we have edited the paragraph to clarify the message and hope the text reads more clearly now.

"2. The data in Figure 6A does not fully support the conclusions. The authors show that upon induction with Dox of the stably transduced cell line T-Rex-292-F12-HA the expression of F12 is induced. Upon infection with a vaccinia virus lacking F12, the authors claim that they co-precipitate a higher amount of A36 with KLC1 upon induction of F12. Based on these data, the authors suggest in the abstract and in Figure 8 that the F12/E2 complex cooperatively enhances the association of A36 with KLC1. However, this conclusion would only be correct if the induction with Dox would not change the expression levels of the Flag-tagged KLC1.
But unfortunately, exactly this part of the blot shown is messed up. If the authors cannot provide a blot with clear data on Flag-tagged KLC1, F12, and A36, the authors need to remove this part of the manuscript. They claim that they have conducted this experiments several times, and even provide a quantitation of such blot data in Fig. 6B. However, without supportive primary data this quantitation is flawed.

Furthermore, without these data, the major conclusion of the manuscript would be lost."

The evidence that the induction of Dox does not change the expression levels of Flag-tagged KLC1 or KLC2 was presented very clearly in the original figure, see Figure 6A left panel (inputs). The levels of KLC1 and KLC2 are definitely not affected by addition of Dox; compare the lanes + and – Dox. So we disagree with this particular statement from the referee.

However, we agree with the referee that for the immunoprecipitated proteins the top panel of the right half of this figure is distorted ("messed up") for the anti-Flag blot. This was simply because of overloading of protein sample. So we have re-run all these samples (inputs and IPs) and now provide clearer blots showing unequivocally that both the total amount and the amount of immunoprecipitated KLC1 and KLC2 are the same in the presence or absence of Dox.

These data therefore fully support the conclusion that the F12/E2 complex enhances or stabilises the association of A36 with KLC.

We might add that we took great care to control for any non-specific effects of Dox treatment on the assay and had even done this following transfection of the Flag-tagged KLCs into a control cell line made using the same plasmid vector but not expressing F12-HA, see Figure 6B panel ii. Again the addition of dox made no difference to the levels of KLCs expressed.

To support the quantitative data presented in Figure 6B we have also added the primary data blots used to generate the quantitative analysis in figures 6Bi and 6Bii as an additional supplemental figure (Supplemental Fig 1A and 1B).

Lastly, we point out that the conclusion of this paper is not based solely based on the results presented in Figure 6. Figure 7 shows clearly a reduction in A36 association with KLC following infection with viruses lacking either F12 or E2, compared to infection with wild type virus.

Minor Comments:

"1. Reference 64 does not provide data showing that pUL36 of herpes simplex virus interacts with KLC. This comment needs to be removed. However, there is newer data deleting potential KLC binding motifs in pUL36 from the Sodeik group in Germany that should be discussed in this context."

We have added a reference from the Sodeik lab describing the mutation of potential WE/D motifs in the HSV pUL36 and reworded the relevant section, instead referring to the pUL36 (VP1/2) of alphaherpesviruses.

____

"2. From which species are the expression constructs for the FLAG-tagged kinesin-light chains? "

____

The KLC alleles used are of murine origin. We have added this information to the materials and methods. These have been described previously (see references given in materials and methods) and the very high level of amino acid identity to human KLC has been discussed in supplemental information in a PLoS Pathogens paper published by our lab (Carpentier et al 2015). A reference to this paper has been added in the relevant section of Materials and Methods.

We hope that these explanations and changes to the manuscript and figures address the issues raised.

With kind regards
Yours sincerely

Geoffrey L Smith

Decision and Reviews

Dear Dr. Smith,

Thank you for submitting your revised manuscript "Vaccinia Virus Proteins A36 and F12/E2 Show Strong Preferences for Different KLC Isoforms" to Traffic. I asked referee 2 to read the revised paper and his/her verbatim comments are appended below. I agree with the referee that you have addressed the concerns raised previously. The referee recommend citing one additional reference. With this very minor revision I would be pleased to accept this paper for publication in Traffic.

Sincerely,

Trina A. Schroer, Ph.D.
Co-Editor

____

Referee's Comments to the Authors

Referee: 2

Comments to the Author
The authors have addressed my comments to their manuscript in their revision.

Yes, they are correct, I have overlooked the loading control in Fig. 6A; my apologies. Nevertheless, it is good that they have improved the other part of Fig. 6A.

As for reference to data from the alphaherpesvirus field; in addition to reference 65 for pseudorabiesvirus, there is also a study for HSV-1 showing by live cell imaging that pUL36 is required for microtubule-mediated egress (Sandbaumhüter et al. 2013) which should be quoted in this context (page 5, right column, middle of first paragraph).
